# Tea Administration Facilitates Immune Homeostasis by Modulating Host Microbiota

**DOI:** 10.3390/nu16213675

**Published:** 2024-10-29

**Authors:** Yihui Wang, Jiayu Zhou, Min Yang, Liying Zhu, Feifei Wang

**Affiliations:** 1 Haide College, Ocean University of China, Qingdao 266100, China; 22220002061@stu.ouc.edu.cn; 2 Key Laboratory of Medical Molecular Virology (MOE/NHC/CAMS), Shanghai Institute of Infectious Disease and Biosecurity, Department of Medical Microbiology and Parasitology, School of Basic Medical Sciences, Shanghai Medical College, Fudan University, Shanghai 200032, China; 23211010059@m.fudan.edu.cn (J.Z.); 23211020038@m.fudan.edu.cn (M.Y.); 21111010094@m.fudan.edu.cn (L.Z.)

**Keywords:** tea, immune homeostasis, microbiota, gut-lung, gut-brain, gut-liver

## Abstract

Tea, derived from the young leaves and buds of the *Camellia sinensis* plant, is a popular beverage that may influence the host microbiota. Its consumption has been shown to promote the growth of beneficial bacterial species while suppressing harmful ones. Simultaneously, host bacteria metabolize tea compounds, resulting in the production of bioactive molecules. Consequently, the health benefits associated with tea may stem from both the favorable bacteria it nurtures and the metabolites produced by these microbes. The gut microbiota plays a vital role in mediating the systemic immune homeostasis linked to tea consumption, functioning through complex pathways that involve the gut–lung, gut–brain, and gut–liver axes. Recent studies have sought to establish connections between tea, its bioactive compounds, and immune regulation via the gut microbiota. In this paper, we aim to summarize the latest research findings in this field.

## 1. Introduction

Tea is one of the most widely consumed beverages worldwide. The tea infusion process involves extracting compounds from tea leaves, which serve as unique ecological niches inhabited by various microbes and possess specific environmental characteristics [1]. Despite the complex nature of the resulting boiled extracts, which include plant resources, metabolites, and components derived from microorganisms [2,3], the primary constituents of tea infusions remain the plant resources sourced from tea leaves.

The diverse categories of tea primarily arise from differences in their manufacturing processes, despite all originating from the *Camellia sinensis* plant [4]. Based on the preparation methods, tea is classified into six major types: non-fermented green tea, partially fermented Oolong tea, fully fermented black tea, non-fermented white tea, post-fermented Pu’erh tea, and lightly fermented yellow tea (Table 1) [5,6,7]. Each type exhibits unique medicinal properties, attributed to its distinct chemical composition (Table 1) [8]. It is important to note that these classifications are general; within each type, numerous variations and subcategories exist. The preparation processes and resulting characteristics can differ based on specific tea varieties, growing conditions, and production methods [9].

Tea is rich in polyphenolic compounds [10], theaflavins, L-theanine, caffeine, and volatile organic substances, which offer a range of health benefits [11]. These compounds contribute to its anti-infection, anti-cancer, antioxidant, and anti-aging properties [12,13,14], while also providing cardiovascular protection and reducing hypertension risk [15,16]. As natural immune enhancers, these tea components play a supportive role in maintaining immune homeostasis in humans [17].

Immune homeostasis is a specific type of homeostasis that maintains overall health by controlling organ function and maintaining immunological well-being [18]. The immune system is composed of specific organs and cells that enable humans to defend against undesired responses and maintain the body homeostasis. Nutrition takes part in the regulation of immunological response by providing necessary nutrients to immune cells. There are a large number of macronutrients, such as some amino acids, cholesterol, and fatty acids, as well as some micronutrients, like minerals and vitamins [19]. They are all very important in the modulation of immune function. For example, fatty acids have many possibilities to modulate immune cell function by influencing their structure, metabolism, and function, acting through surface proteins (G-protein-coupled receptors: GPRs), nuclear receptors, or membrane transporters [20]. 

Tea contains functional ingredients that may enhance protection against various health threats, including immune system support and the modulation of specialized immune cells [17,21,22]. This review examines recent research on tea’s impact on immune homeostasis, with a particular focus on the roles of the gut microbiota and other influencing factors.

**Table 1:** Representative tea and characteristics.

Tea	Fermentation	Main Components	Example
Green Tea	It undergoes minimal or no fermentation, and is heated rapidly to halt oxidation, preserving its green color and fresh flavor [23].	Its polyphenolic compounds are mainly flavonoids, flavanols, phenolic acids, and the like [24,25]. It also contains caffeine, amino acids, and vitamins.	Longjing Tea [26] (Hangzhou, China)
Oolong Tea	It undergoes partial fermentation, falling between green and black tea in terms of oxidation [27].	Its specific Oolong tea polyphenol is oolonghomobisflavan B [28,29]. Its composition is affected by the fermentation process [30].	Tie Guan Yin Tea [31] (Fujian, China)
Black Tea	It undergoes full fermentation. The leaves are oxidized until they turn dark brown, which results in a robust flavor.	It has the same origin as green tea and the main polyphenolic compounds in different amounts [32]. Most of the catechins are oxidized and polymerized [33]. Other compounds are theaflavins, theabrownins, and thearubigins.	Darjeeling Tea [34] (India)
White Tea	It undergoes the least amount of processing and fermentation among teas. The young buds and leaves are simply steamed or fired to dry them [35].	It contains high levels of theabrownins, soluble sugar, and flavonoids and 25 aroma compounds [36]. The contents of theanine, catechins, theasinesins, 3 proanthocyanidins, and phenolic acids decreased significantly during the withering period [37].	Bai Hao Yin Zhen [35,38] (Fujian, China)
Pu’erh Tea	It undergoes post-fermentation, where the tea is stored and aged to develop a unique flavor and aroma [39]. This process can last for years.	It is rich in microbial communities and metabolites that develop during pile fermentation [40]. It also contains enriched theabrownins and phenolic acids such as gallic acid [41].	Pu’erh Tea [40,42] (Yunnan, China)
Yellow Tea	It undergoes a brief period of fermentation followed by a unique “yellowing” process where the leaves are covered to induce non-enzymatic oxidation [43].	It contains high contents of theanine, glutamic acid, aspartic acid, and flavonoids [44]. Pheophorbides, carotenoids, thearubigins, and theabrownins are the major pigments contributing to the “three yellows” appearance [45,46]. The yellowing process may alter the composition and produce unique flavors [46].	Mengding Huangya [46] (Sichuan, China)

## 2. Microorganisms Are Involved in Metabolism of Tea Components

Fermentation is a crucial process in tea production, enhancing its sensory qualities, nutritional value, and health benefits. This process involves physical, biochemical, and microbial changes, with the specific microbial species significantly influencing tea quality. For instance, *Eurotium cristatum* (*E. cristatum*) facilitates the conversion of phenolics found in unfermented green tea, improving color, taste, and flavor in the resulting fermented tea [47,48]. Lee et al. investigated the in vitro interactions between 37 different human gut microbiota strains and green tea polyphenols, the primary bioactive compounds in tea, demonstrating bidirectional effects. Mass spectrometry analysis of culture broth extracts showed that *Adlercreutzia*, *Eggerthella*, and *Lactiplantibacillus plantarum* (*L. plantarum*) KACC11451 promoted the C-ring opening reaction in green tea catechins. Additionally, *L. plantarum* hydrolyzed catechin galloyl esters, producing gallic acid and pyrogallol, and converted flavonoid glycosides into their aglycone forms [49]. Conversely, these biotransformed polyphenols enhanced antioxidant bioactivity and inhibited most species within the phyla *Actinobacteria*, *Bacteroides*, and *Firmicutes*, with the exception of the genus Lactobacillus [49]. This finding underscores the complex interactions between dietary polyphenols and the gut microbiome.

Moreover, the microbial components in tea are closely linked to the metabolism of tea components in vivo. Zhu et al. isolated a strain of golden flower fungus, identified as *Aspergillus cristatus*, from Fu brick tea and found that its polysaccharides reduced obesity in rats. These polysaccharides altered the gut bacterial composition, increased fecal short-chain fatty acids, and elevated bile acid levels in the serum, liver, and feces [48,50].

## 3. Tea Consumption Regulates the Gut Microbiota

Ingested tea, like other beverages, is primarily absorbed through the digestive tract, making the gut a critical site of action for its bioactive compounds [51]. Numerous reports have demonstrated that tea components have the potential to modulate the gut microbiome [52]. For instance, both black and green tea have been shown to enhance populations of beneficial bacteria such as *Allobaculum, Lactobacillus*, and *Turicibacter* in hyperglycemic mice. This increase occurs alongside a reduction in harmful or conditionally pathogenic bacteria, particularly those in the *Clostridiales* and Bacteroides groups [53]. One specific compound, epigallocatechin-3-gallate (EGCG) from green tea, has demonstrated a notable ability to promote the growth of *Akkermansia muciniphila*, a beneficial microorganism associated with reduced obesity and improved metabolic health.

Mechanistically, co-administration of green tea powder and *Lactobacillus plantarum* has been shown to significantly increase both *Lactobacillus* abundance and overall bacterial diversity in the intestine. *Lactobacillus* species function primarily as probiotics, engaging with the gut mucosal environment to induce beneficial physiological responses. This interaction not only supports gut health but also facilitates an increased presence of *Akkermansia* species [54]. The combination has also been shown to effectively reduce body fat content as well as hepatic accumulation of triacylglycerol and cholesterol [55]. Thus, green tea may serve as a potential prebiotic agent for *Akkermansia*, with possible applications in treating metabolic syndromes [56,57]. Interestingly, black tea has been shown to have a superior effect compared to green tea in regulating glycolipid metabolism [53].

To validate the pivotal roles of tea polyphenols in modulating the gut microbiota, experiments were conducted where decaffeinated green tea and black tea were administered to mice on a high-fat/high-sucrose diet [58,59]. These interventions not only inhibited weight gain in the mice but also resulted in a significant decrease in the abundance of *Firmicutes* in the cecum of those consuming an obesogenic diet, while simultaneously increasing *Bacteroidetes* levels [58]. The relative abundances of specific bacterial genera, including *Blautia*, *Bryantella*, *Collinsella*, *Lactobacillus*, *Marvinbryantia*, *Turicibacter*, *Barnesiella*, and *Parabacteroides*, were significantly associated with weight loss induced by tea extracts [58]. Notably, black tea extracts increased the relative abundance of *Pseudobutyrivibrio* and promoted the intestinal production of short-chain fatty acids (SCFAs) [58]. Both black tea and green tea extracts induced weight loss, which was associated with changes in microbiota composition and increased phosphorylation of hepatic AMP-activated protein kinase [58].

Furthermore, metabolites and products derived from microorganisms in tea play a crucial role in regulating host immune homeostasis. For instance, intracellular polysaccharides isolated from *Aspergillus cristatus* (MK346334, NCBI) in Fuzhuan brick tea have demonstrated immunomodulatory effects in mice [50]. The immunomodulation may result from the maintenance of gut homeostasis through the modulation of the gut microbiota, increased production of SCFAs, and enhanced intestinal barrier function [50].

Overall, tea effectively regulates the gut microbiota by altering its composition, promoting the growth of beneficial strains, and inhibiting the proliferation of harmful ones.

## 4. Tea Consumption Also Regulates Oral Microbiome

Green tea polyphenols (GTPs) have been shown to induce changes in both the oral and gut microbiomes. Notably, intestinal colonization by bacteria of oral origin is closely associated with colorectal carcinogenesis [60,61]. GTPs modify the salivary microbiota and reduce the abundance of functional pathways linked to carcinogenesis, including by lowering fecal levels of *Fusobacterium*, which adheres to, invades, and promotes oncogenic and inflammatory responses in colorectal cancer cells through its unique FadA adhesin [61]. Notably, in individuals consuming green tea liquid, both the *Lachnospiraceae* family and the B/E ratio (the ratio of *Bifidobacterium* to *Enterobacteriaceae*, a marker of colonization resistance) are negatively correlated with the presence of oral-like bacterial networks in fecal samples [60].

Mechanism-focused studies consistently show that tea consumption can alter the composition of oral bacteria in humans [62]. However, individual responses vary, particularly regarding how green tea influences miRNA expression in oral epithelial cells. This suggests that while tea generally impacts the oral microbiota, its effect on specific molecular markers within the oral cavity may depend on individual factors [63]. In contrast, analysis of the microbiome on cancer-prone lingual mucosa revealed significant shifts in the relative abundance of *Streptococcus*, *Staphylococcus*, and other genera following green tea exposure [63].

Research in rodents consuming GTPs has revealed significant changes in epithelial gene expression [64]. Notably, GTPs are not readily absorbed by the digestive tract epithelium but instead are metabolized by gut and oral microbial enzymes [49]. This metabolic process can alter their absorption and functionality, thereby impacting their bioactivity [64]. This variability explains inconsistencies observed in RNA expression changes in human oral epithelium following green tea consumption [64]. Since each individual has a unique gut and oral microbiome, the levels of bacteria capable of metabolizing polyphenols can vary widely. The consistency of tissue responses to green tea in rodent models allows for selecting a dose level that impacts tumor rates [64]. Consequently, determining an optimal green tea dose for humans would require an understanding of each individual’s unique gut and oral microbiome [64].

## 5. Tea Modulates the Immune Homeostasis via the Gut Axes

### 5.1. Gut–Lung Axis

Certain lung diseases, such as coronavirus disease 2019 (COVID-19), disrupt the delicate balance of intestinal microecology, often resulting in intestinal complications [65,66]. This highlights the direct immunological interaction between the lungs and the gut.

Tea polyphenol compounds, known for their antiviral and prebiotic properties, show promise in mitigating lung-related diseases through the gut–lung axis [21]. They can help restore microbial flora imbalances, reduce the occurrence of cytokine storms, and potentially prevent COVID-19 infections. Therefore, tea polyphenol compounds represent a valuable resource for developing novel antiviral drugs, combining high efficacy with low toxicity (Table 2) [21,65].

Furthermore, mice exposed to particulate matter exhibited oxidative stress and inflammation in their lungs. However, a daily intake of black tea infusion significantly mitigated these effects in a concentration-dependent manner [67]. Notably, the ethanol precipitate fraction of the tea infusion was identified as the primary contributor to these protective effects. Fecal microbiota transplantation studies reveal that tea infusion and its fractions can reshape the gut microbiota in mice, directly alleviating lung injury through the gut–lung axis [67]. Among the various gut microbes, the *Lachnospiraceae*_NK4A136 group emerged as a core contributor to this protective mechanism [67].

### 5.2. Gut–Brain Axis

Evidence increasingly points to complex interactions between the gastrointestinal tract and central nervous system, encompassing biochemical signaling, microbiota modulation, hypothalamic–pituitary–adrenal (HPA) axis activity, and bidirectional communication [68]. For instance, theaflavins, compounds formed through the enzymatic oxidation of catechins during tea production, have shown effectiveness in enhancing behavioral function through the microbiota–gut–brain axis [69]. These compounds promote gut homeostasis by restructuring the gut microbiota and influencing key metabolites, such as short-chain fatty acids and essential amino acids, which in turn upregulate neurotrophic factors in the brain [69]. Notably, eliminating the gut microbiota with antibiotics partially reduces the neuroprotective effects of theaflavins. Correlation analyses indicate a positive association between behavioral improvements and a decrease in the gut microbiota such as *Bacteroidetes* and *Lachnospiraceae*, alongside an increase in microbiota metabolite levels (Table 2) [69]. Similar regulatory mechanisms are observed with tea bioactive extracts in managing polystyrene microplastic (PS-MP)-related anxiety. PS-MPs compromise intestinal barrier integrity through the gut microbiota, raising peripheral inflammatory cytokines and triggering anxiety-like behaviors. Furthermore, EGCG (CAS#: 989-51-5), the primary bioactive component in green tea, demonstrates anxiolytic effects through the gut–brain axis by optimizing gut microbiota composition, and inhibiting the hippocampal TLR4/MyD88/NF-κB signaling pathway [70].

**Table 2:** Tea regulates immune responses in distant organs via gut microbiota.

Axis	Representative Disease	Experimental Model	Administered Components and Dosage	Administration Route	Related Microbes	Potential Mechanisms
Gut–lung [71]	COVID-19	Male C57BL/6 J mice (3-week-old)	EGCG 10 mg/kg	Free access	*Lactobacillus* and *Bifidobacterium* [72]	EGCG, via activating Nrf2, can suppress ACE2 receptors and TMPRSS2 during SARS-CoV-2 infection [73].
Gut–brain [69]	Aging-associated cognitive dysfunction	Eight-week-old male ICR mice	Theaflavins 50 mg/kg	Oral gavage	*Bacteroidetes* and *Lachnospiraceae*	TF treatment maintained gut homeostasis by improving antioxidant ability, maintaining the integrity of the intestinal mucosal barrier, restructuring the gut microbiota, and altering microbiota metabolites, and upregulated brain neurotrophic factors as well as alleviated cognition and spatial memory impairments.
Gut–liver [74]	Hypercholest-erolemia	Male C57BL/6 J mice (3-week-old)	Theabrownins 225 mg/Kg/day	Free access	*Lactobacillus*, *Bacillus*, *Streptococcus* and *Lactococcus*	TBs increase the levels of ileal conjugated bile acids (BAs) which, in turn, inhibit the intestinal FXR-FGF15 signaling pathway, resulting in increased hepatic production and fecal excretion of BAs, reduced hepatic cholesterol, and decreased lipogenesis.

Tea polyphenol compounds, important functional compositions in tea, actively influence the composition and functionality of the intestinal microbiota. As reported, disruptions in the circadian rhythm have been linked to various human metabolic disorders, and these rhythms are observed not only in the hypothalamus, a crucial brain region, but also within the intestinal microbiota. Research suggests that tea polyphenol compounds may help regulate circadian-rhythm-related diseases by modulating the intestinal microbiota [75]. Moreover, numerous studies have highlighted the neuroprotective and neuro-reparative properties of tea polyphenol compounds, which have been shown to influence neurotransmission and behavior through the microbe-gut–brain axis, often linked to neuropsychiatric disorders [76]. Thus, tea polyphenol compounds interact with the gut microbiota, producing intermediate metabolites that help regulate the composition and functionality of intestinal flora. Additionally, these compounds affect appetite control through the gut–brain axis, offering valuable nutritional insights for dietary preferences [77,78].

Similarly, gut microbiota dysfunction is closely associated with depression. Studies indicate that jasmine tea can alleviate depressive-like behaviors and elevate neurotransmitter levels in rats under chronic unpredictable mild stress. Further analysis revealed correlations between specific alterations in the gut microbiota (including *Patescibacteria*, *Firmicutes*, *Bacteroidetes*, *Spirochaetes*, *Elusimicrobia*, and *Proteobacteria*) and depressive-related biomarkers (such as BDNF, GLP-1, and 5-HT) in the hippocampus and cerebral cortex [79]. Additionally, the intake of Pu-erh tea significantly modulated the gut microbiome, particularly enhancing the Bifidobacterium population, and regulated the metabolism of SCFAs. This beneficial change contributed to reducing blood–brain barrier damage and alleviating neuroinflammation associated with depression-like behaviors by inhibiting the MyD88/NF-κB pathways [80].

Depression and anxiety, as major public health issues, have gained growing interest from researchers in food science and nutrition. Dietary natural products and nutrients, such as tea and dietary fiber, are increasingly recognized as crucial factors in the prevention and management of these conditions. Epidemiological studies have consistently demonstrated that regular tea consumption can significantly lower the risk of depression. The bioactive compounds found in tea, including L-theanine, catechins, and tea pigments, demonstrate antidepressant effects by inhibiting hyperactivity of the hypothalamic–pituitary–adrenal axis, enhancing the diversity of intestinal flora, and promoting the activity of the microbial-gut–brain axis [81]. These compounds may exert their effects through mechanisms that modulate the microbiota–gut–brain axis, suppress hypothalamic–pituitary–adrenal axis hyperactivity, and regulate levels of monoamine neurotransmitters [81]. Despite their potential benefits, the limited bioavailability of these natural compounds in tea restricts their effectiveness in managing depression. To overcome this challenge, emerging technologies like metabolomics, proteomics, and genomics, as well as nano-encapsulation techniques, can be utilized to enhance the stability and bioavailability of tea’s active ingredients while minimizing potential biotoxicity [82].

### 5.3. Gut-Liver Axis

Tea is known for its antioxidant, anti-inflammatory, and hepatoprotective properties. Kombucha, a fermented nonalcoholic tea beverage created from a symbiotic culture of bacteria and yeasts, reduces oxidative stress and inflammation, enhances liver detoxification, and alleviates intestinal dysbiosis. Additionally, it aids in managing obesity and its associated comorbidities while modulating the gut microbiota in vivo [83]. Matcha green tea, abundant in tea polyphenols, theanine, and caffeine, is recognized for its potential to alleviate metabolic disorders. It effectively regulates glucose, lipid, and bile acid metabolism, positioning it as an effective nutritional strategy for mitigating obesity and related metabolic disorders through the gut–liver axis [84].

The bioactive compounds in tea extracts are essential for restoring liver homeostasis. Notably, theabrownin, a highly active and abundant pigment found in Pu-erh tea, significantly influences the gut microbiota by suppressing microbes associated with bile salt hydrolase activity. Moreover, theabrownin increases the levels of ileal conjugated bile acids, which inhibits the intestinal FXR-FGF15 signaling pathway. This is accompanied by an upregulation of enzymes involved in the alternative bile acid synthetic pathway, leading to increased production of hepatic chenodeoxycholic acid, activation of hepatic FXR, and hepatic lipolysis (Table 2) [74]. Another bioactive compound, EGCG in 50 mg/kg (dissolved in PBS, Cat#: S25528, Source Leaf Biological Technology, Shanghai, China), has demonstrated beneficial effects on liver health by enhancing the abundance of probiotics and effectively suppressing polystyrene microplastic (MP)-induced colonic inflammation [85]. EGCG would alleviate MP-induced systemic and hepatic inflammation, fibrosis, and alterations in liver metabolome, positioning it as a potential preventive strategy against these adverse health outcomes [85]. Furthermore, EGCG promotes liver health by increasing antioxidant enzyme activity and modulating the Nrf2 and TLR4/NF-κB pathways, thereby reducing oxidative stress and inflammation caused by hepatotoxin thioacetamide [85]. These antioxidative and anti-inflammatory effects, mediated through the microbiota–gut–liver axis, suggest that EGCG has potential therapeutic value in treating hepatic encephalopathy [86].

Gut microbiota dysbiosis is a key factor in the pathogenesis of alcoholic fatty liver disease (AFLD). Studies have shown that tea supplementation, particularly oolong and dark tea, significantly mitigates liver steatosis, reduces oxidative stress and inflammation, and modulates the gut microbiota in mice chronically exposed to alcohol. Further analysis identified specific bacteria, such as *Bacteroides*, *Alloprevotella*, and *Parabacteroides*, as closely associated with AFLD, highlighting their potential role in the disease process. Thus, the phytochemical components in tea extracts contribute to their preventive effects against AFLD [87]. Dark tea exhibits greater potential in regulating lipid metabolism compared to other tea varieties, with theabrownin identified as a key contributor to its bioactivity. Theabrownin has demonstrated significant preventive and therapeutic effects against non-alcoholic fatty liver disease (NAFLD) and obesity by modulating serotonin levels and related signaling pathways via the gut microbiota. Moreover, the gut microbiota and theabrownin work synergistically to alleviate NAFLD and obesity, positioning theabrownin as a promising therapeutic agent for these conditions [88].

The administration of a water extract from green tea has demonstrated significant efficacy in alleviating alcohol-induced intestinal inflammation and microbiota imbalances, while concurrently restoring intestinal barrier function. Furthermore, the potential incorporation of green tea into long-term nutritional regimens indicates its promise as a preventive measure against alcohol-related health issues [89].

While researchers initially focused on the biological activity of tea infusions, green tea leaf powder also exhibits substantial prebiotic potential due to its high dietary fiber content. It helps prevent dyslipidemia by modulating hepatic mRNA expression and promoting the growth of beneficial gut bacteria while inhibiting harmful bacteria in mice fed a high-fat diet. These alterations enhance lipid metabolism and reduce systemic inflammation, likely through the reprogramming of the gut microbiota [90].

## 6. Conclusions

**Figure 1:**
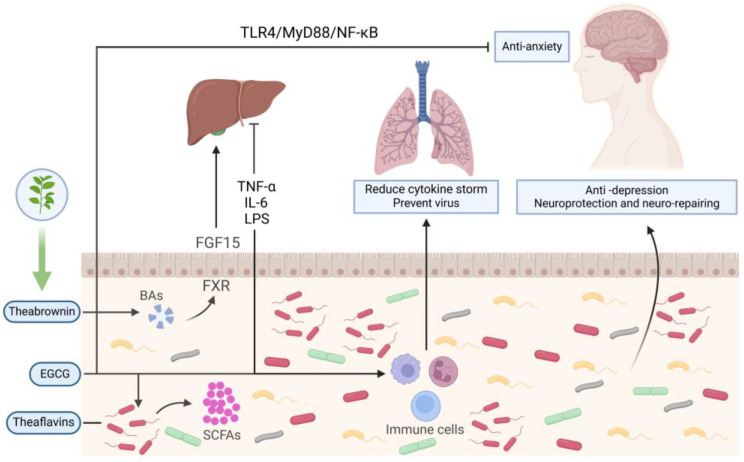
Tea regulates intestinal microbiota and maintains immune homeostasis through gut–liver axis, gut–lung axis, and gut–brain axis. EGCG, epigallocatechin-3-gallate; BAs, bile acids; SCFAs, short-chain fatty acids. Created with BioRender.com.

In summary, tea consumption has a significant role in modulating the composition and functionality of the host microbiota, particularly in the gut microbiome. This modulation allows the bioactive compounds in tea and the metabolites produced by gut microorganisms to help maintain immune homeostasis and potentially alleviate immune-related disorders. Furthermore, tea administration can aid in combating lung diseases, such as infectious diseases, via the gut–lung axis; alleviate nervous system disorders through the gut–brain axis; and improve liver-related diseases via the gut–liver axis (Figure 1).

However, tea’s effects are not solely limited to regulating the host microbiota. Tea components also have the capacity to directly regulate immune activity in lymphocytes and induce the production of effector cytokines, further contributing to the maintenance of host immune homeostasis. Since some diseases are systemic, single-target intervention is proven to be ineffective. Additionally, the pleiotropic natural products in tea are multi-targeting. Thus, network pharmacology approaches should be used in researching of bioactivity of tea components since they address the ability of these natural products to target numerous proteins or networks involved in a disease. Given the numerous bioactive compounds present in tea, it is promising to identify those that are most beneficial for human health and optimize their extraction processes to enhance their efficacy.
